# Brazilian green propolis water extract up-regulates the early expression level of HO-1 and accelerates Nrf2 after UVA irradiation

**DOI:** 10.1186/s12906-015-0945-4

**Published:** 2015-11-26

**Authors:** Yuichi Saito, Kazuhiro Tsuruma, Kenji Ichihara, Masamitsu Shimazawa, Hideaki Hara

**Affiliations:** Department of Biofunctional Evaluation, Molecular Pharmacology, Gifu Pharmaceutical University, 1-25-4 Daigakunishi, Gifu, 501-1196 Japan; Nagaragawa Research Center, Api.Co., Ltd., 692-3 Nagara, Gifu, 502-0071 Japan

**Keywords:** Propolis, Nrf2, HO-1, Skin fibroblast, UVA

## Abstract

**Background:**

Exposure to ultraviolet A (UVA) irradiation is the major cause of human skin aging. Suppression of UVA irradiation-induced skin fibroblast cell damage protects the skin against aging. An oxidative stress response transcription factor nuclear factor-(erythroid-derived 2)-related factor 2 (Nrf2) has an important role as a cytoprotective system against oxidative stress in the human skin and other organs. Propolis has been commonly used as a traditional medicine since ancient times. The water extract of propolis (WEP) mainly contains caffeoylquinic acids. In our previous study, we reported that WEP and its major constituents protected immortalized human skin fibroblast cells (NB1-RGB) against UVA irradiation-induced cell death. In this study, we examined the mechanism of WEP-mediated skin protection and the possible involvement of Nrf2/antioxidant response element (ARE) pathways.

**Methods:**

Brazilian green propolis was used in the present study (Minas Gerais State, Brazil), *Baccharis dracunculifolia* is its main source. The *Baccharis* propolis was extracted with water at 50 °C to yield water extract. The NB1-RGB cell cultures were incubated for 23 h. After replenishing the medium, WEP or its constituents were added to the cell cultures. After 1 h, the cells were exposed to 10 J/cm^2^ of UVA light (365 nm UVA light source, CL-1000 L UV Closslinkers, Ultraviolet Products Ltd., Cambridge, UK). Heme oxygenase-1 (HO-1) expression levels in NB1-RGB cells were evaluated using western blotting. Nrf2 nuclear translocation changes in NB1-RGB cells were indicated using immunostaining.

**Results:**

We demonstrated that WEP pretreatment up-regulated HO-1 expression level after UVA irradiation at earlier time points than vehicle pretreatment did, and three main constituents of WEP showed similar effects. Furthermore, WEP pretreatment also accelerated Nrf2 nuclear translocation after UVA irradiation.

**Conclusions:**

Our findings indicated that WEP acts as an early inducer of HO-1 and rapid activator of Nrf2 to protect against UVA-induced oxidative stress.

## Background

Human skin comprises the epidermis and dermis, which is mainly composed of collagen, elastin, and hyaluronan synthesized by skin fibroblasts. These proteins maintain skin elasticity and prevent skin wrinkling. Skin fibroblasts are also present in the dermis and control the production of components of the extracellular matrix [1]. Ultraviolet A (UVA) radiation occurs at the longest wavelengths (320–400 nm) of the UV spectrum and is harmful to the human skin. UVA produces reactive oxygen species (ROS) in human skin fibroblast, and ROS induces oxidative stress leading to cell death [2, 3]. Fibroblast cell death decreases collagen, elastin, and hyaluronan synthesis, which results in skin aging. UVA has the highest permeability potential of all the UV wavelengths. Therefore, most of the UVA radiation permeates the ozone layer, reaches the skin, and is the major cause of human skin photoaging [4]. To protect skin from photoaging, it is important to suppress UNA irradiation-induced fibroblast cell death.

An oxidative stress response transcription factor nuclear factor-(erythroid-derived 2)-related factor 2 (Nrf2) is known to be one of the major cytoprotective systems against oxidative stress in the human skin and other organs [5]. Under normal conditions, Nrf2 is inactivated by interaction with the Kelch-like ECH-associated protein 1 (Keap1) because it is continuously degraded by ubiquitin-mediated proteolysis. Under oxidative stress condition such as those induced by UVA irradiation, chemical oxidants (e.g., cadmium or sodium arsenite), and high glucose exposure, Nrf2 detaches from Keap1 and translocates to the nucleus to activate the antioxidant response element (ARE). As a result, antioxidant response proteins such as heme oxygenase-1 (HO-1) and nicotinamide adenine dinucleotide phosphate [NAD(P)H] quinone oxidoreductase 1 (NQO-1) are induced and mediate cytoprotective effects [6–8]. Recently, an Nrf2 activator, bardoxolone methyl, was developed for treatment of chronic kidney disease [9, 10]. Moreover, Nrf2 activators have potential for treatment of various pathological conditions which relate with oxidative stress.

Propolis has been commonly used as a traditional medicine since ancient times. It is prepared from a mixture of waxes collected by honeybees from the resinous sap of certain trees and flowers. The properties and constituents of propolis differ and depend on its geographical origin [11]. Brazilian green propolis has been reported to have biological activities such as antibacterial [11–13], anti-inflammatory [14], antioxidative [15, 16], and antitumor [16, 17]. The solvents used for extraction, including water and ethanol, also influence the biological activities of propolis. Ethanol extract of propolis (EEP) mainly contains cinnamic acid derivatives such as artepillin C or baccharin. In contrast, water extract of propolis (WEP) mainly contains caffeoylquinic acids [18]. In our previous study, we reported that WEP and its major constituents protect immortalized human skin fibroblast cells (NB1-RGB) from UVA irradiation-induced cell death [19]. Furthermore, we also reported that UVA irradiation-induced ROS production is suppressed by pretreatment with WEP. In this study, we investigated the skin protectant effects of propolis focusing on possible mediation via the Nrf2/ARE pathway. Our findings indicated that WEP acts as an early inducer of HO-1 and rapid activator of Nrf2 to protect against UVA-induced oxidative stress.

## Methods

### WEP and its constituents

Brazilian green propolis was used in the present study (Minas Gerais State, Brazil), *Baccharis dracunculifolia* is its main source. The *Baccharis* propolis was extracted with water at 50 °C to yield water extract. The main constituents of WEP were previously reported [18]. WEP and 3,4-di-O-caffeoylquinic acid (3,4-CQA), 3,5-di-O-caffeoylquinic acid (3,5-CQA), *p*-coumaric acid (*p*-CA), and chlorogenic acid (CGA) were a gift from the Api Co., Ltd., (Gifu, Japan) (Table 1).

**Table 1:** HPLC results of WEP used in this study

*p*-coumaric acid (*p*-CA)	34.7 mg/g
chlorogenic acid (CGA)	19.9 mg/g
3,4-di-O-caffeoylquinic acid (3,4-CQA)	69.7 mg/g
3,5-di-O-caffeoylquinic acid (3,5-CQA)	58.5 mg/g

### Cell culture

Normal human skin fibroblast (NB1-RGB) cells were purchased from the Riken Bioresource Center Cell Bank (Tsukuba, Japan). The cells were maintained in Dulbecco’s modified Eagle medium (DMEM, Nacalai Tesque Inc., Kyoto, Japan) containing 10 % fetal bovine serum (FBS, Sigma-Aldrich, St. Louis, MO, USA), 100 U/mL penicillin (Meiji Seika Kaisha Ltd., Tokyo, Japan), and 100 μg/mL streptomycin (Meiji Seika) under a humidified atmosphere of 5 % CO_2_ at 37 °C. The cells were passaged by trypsinization every 3–4 days. The cells were incubated in phenol red-free DMEM (Nacalai Tesque Inc.) containing 1 % FBS for 1 h before beginning the UVA irradiation exposure.

### Exposure of NB1-RGB to UVA irradiation

The NB1-RGB cell cultures were seeded at a density of 2.5 × 10^4^ cells/well in 24-well plates for western blot analysis or glass chamber slides (Laboratory-Tek, Thermo Fisher Scientific, Rockford, IL, USA) for immunostaining, and incubated for 23 h. After replenishing the medium, WEP or its constituents were added to the cell cultures. After 1 h, the cells were exposed to 10 J/cm^2^ of UVA light (365 nm UVA light source, CL-1000 L UV Closslinkers, Ultraviolet Products Ltd., Cambridge, UK). The UVA light was positioned above the 24-well plate at a fixed distance of 11.5 cm. Control cells were treated with vehicle and incubated in the same UVA chamber covered with aluminum foil to avoid UVA irradiation, after which all the cells were returned to the CO_2_ incubator.

### Western blot analysis

The NB1-RGB cells were washed with PBS, lysed with radioimmunoprecipitation assay (RIPA) buffer (Sigma-Aldrich) containing 1 % protease inhibitor cocktail and 1 % of the phosphatase inhibitor cocktails 2 and 3 (Sigma-Aldrich), and harvested. Lysates were centrifuged at 12,000 × *g* for 20 min at 4 °C. Protein concentrations were measured using a BCA protein assay kit (Thermo Fisher Scientific) with bovine serum albumin (BSA) as a standard. Lysates were mixed with sample buffer containing 10 % 2-mercaptoethanol, and subjected to 10 % sodium dodecyl sulfate-polyacrylamide gel electrophoresis (SDS-PAGE). The separated proteins were then transferred onto a polyvinylidene difluoride membrane (Immunobilon-P, Merck KGaA, Darmstadt, Germany). The membranes were incubated with the following primary antibodies: HO-1 rabbit polyclonal (1:1000, Merck KGaA), NAD(P)H dehydrogenase, quinone 1 (NQO1) rabbit polyclonal (1:500, Merck KGaA), and β-actin mouse monoclonal (Santa Cruz Biotechnology Inc., Santa Cruz, CA, USA). Then, the membrane was further incubated with the secondary antibodies including horseradish peroxidase (HRP)-conjugated goat anti-rabbit IgG (1:2000, Thermo Fisher Scientific) and HRP-conjugated goat anti-mouse IgG (1:2000, Thermo Fisher Scientific). The immunoreactive bands were visualized using Immunostar-LD (Wako, Osaka, Japan) and an LAS-4000 luminescent image analyzer (Fuji Film Co., Ltd., Tokyo, Japan). β-actin was used as the loading control.

### Immunostaining

The NB1-RGB cells were fixed with 4 % paraformaldehyde for 15 min, blocked with 3 % goat serum for 30 min, and then incubated overnight at 4 °C with the primary antibody Nrf2 rabbit polyclonal (1:50, Santa Cruz Biotechnology Inc.). After washing the cells, they were incubated for 1 h with secondary antibody (Alexa Fluoro 488 goat anti-rabbit IgG, Thermo Fisher Scientific). The cells were then washed again and counter-stained with Hoechst 33342 (Thermo Fisher Scientific). Images were acquired using a confocal fluorescence microscope (Olympus, Tokyo, Japan).

### Nuclear and cytoplasmic extraction

The NB1-RGB cell cultures were seeded at a density of 3.0 × 10^5^ cells in 10 cm dish. Sampling and extraction are followed by ProteoExtract® Subcellular Proteome Extraction Kit (Merck KGaA).

### Statistical analysis

Data are presented as means ± standard error of the mean (SEM). Statistical comparisons were performed using the Tukey’s test, Student’s *t*-test, or Dunnett’s test. A value of *P* < 0.05 was considered statistically significant.

## Results

### WEP induced early HO-1 up-regulation in NB1-RGB cells only after UVA irradiation

**Fig. 1:**
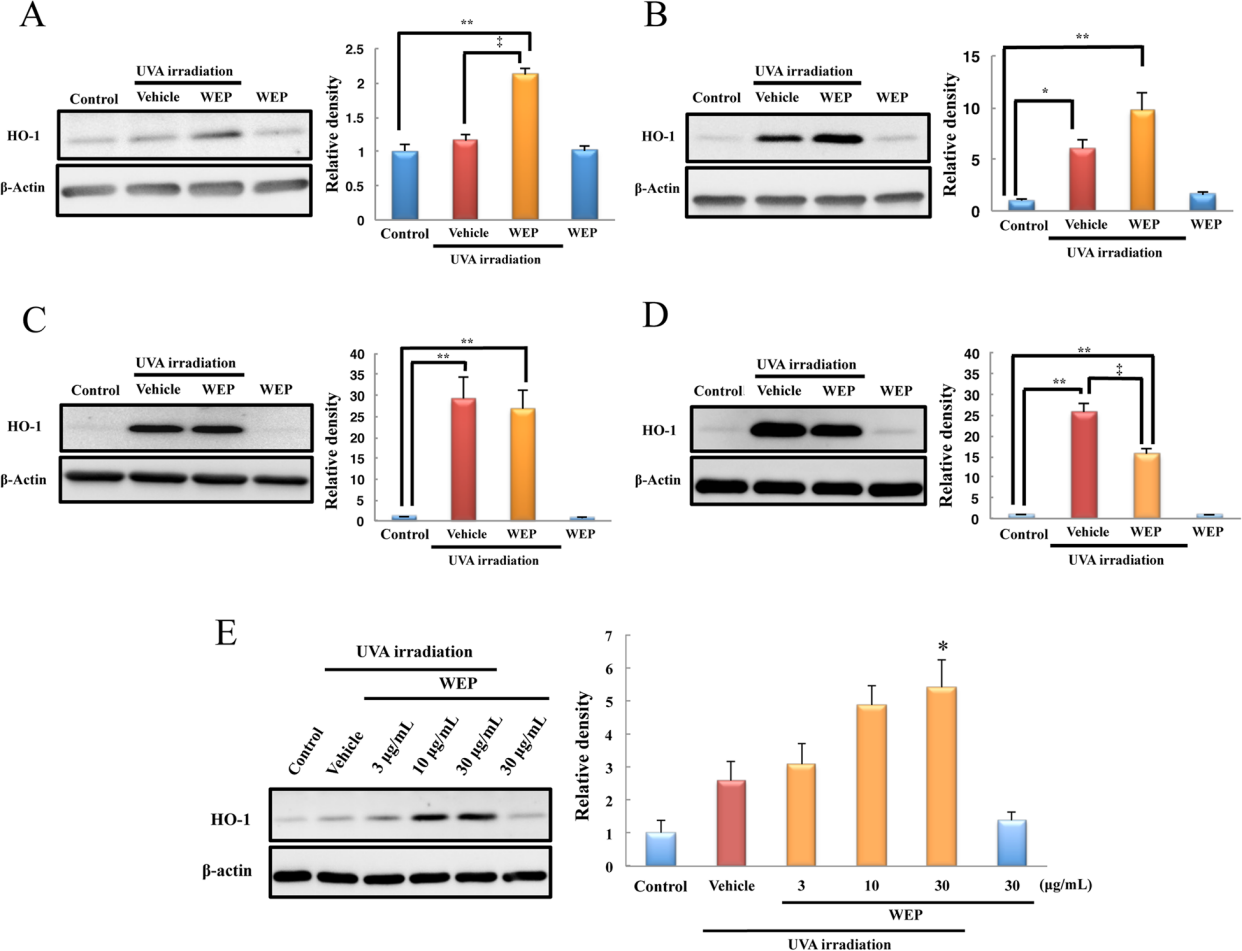
WEP induced HO-1 up-regulation at early time points only after UVA irradiation. **a**–**d** HO-1 expression level in NB1-RGB cells was evaluated using western blotting at each time point after initiating UVA irradiation (**a**) 3, (**b**) 6, (**c**) 12, and (**d**) 24 h. At all time points, WEP was applied at a concentration of 30 μg/mL. Mean ± SEM (*n* = 5–6), ^*^ *P* < 0.05 vs. control, ^**^ *P* < 0.01 vs. control, and ^‡^ *P* < 0.01 vs. vehicle (Tukey’s test). (**e**) HO-1 expression level in NB1-RGB cells was evaluated using western blotting. WEP was applied at 3, 10, or 30 μg/mL 3 h after initiating UVA irradiation. Mean ± SEM (*n* = 6), control vs. vehicle (Student’s *t*-test), ^*^ *P* < 0.05 vs. vehicle (Dunnett’s test)

First, we examined the time course of HO-1 protein expression after UVA irradiation; 3 h after UVA irradiation, HO-1 was significantly up-regulated only in the group treated with both WEP and UVA (WEP-UVA group, Fig. 1a). HO-1 up-regulation was also observed in the UVA-treated group at 6 h (Fig. 1b), and persisted in both groups for up to 12 h (Fig. 1c). At 24 h, HO-1 expression in the WEP-UVA group significantly decreased more than that of the UVA-treated group did (Fig. 1d). The early HO-1 expression level increased concentration-dependently (Fig. 1e).

### WEP constituents showed early up-regulation of HO-1

**Fig. 2:**
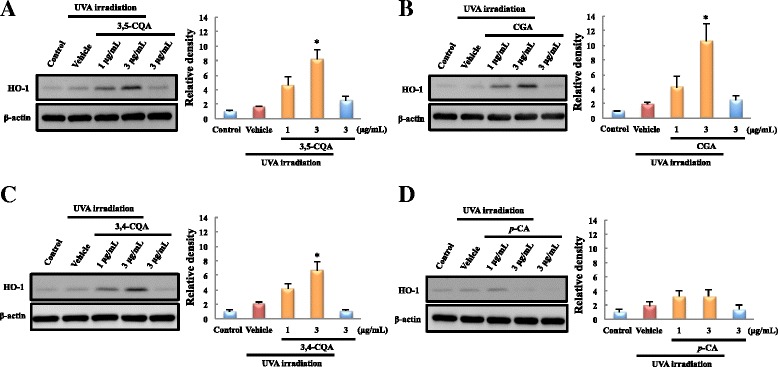
Three main constituents of WEP induced HO-1 expression at early time points after UVA irradiation. HO-1 expression level in NB1-RGB cells was evaluated using western blotting 3 h after initiating of UVA irradiation. **a** 3,5-Di-O-caffeoylquinic acid (3,5-CQA), (**b**) chlorogenic acid (CGA), (**c**) 3,4-di-O-caffeoylquinic acid (3,4-CQA), and (**d**) *p*-coumaric acid (*p*-CA). Each constituent was applied at 1 or 3 μg/mL. Mean ± SEM (*n* = 3–4), ^*^ *P* < 0.05 vs. vehicle (Tukey’s test)

WEP was previously reported to contain four main constituents including 3,4-CQA, 3,5-CQA, *p*-CA, and CGA [18]. Therefore, we examined the effect of these constituents on HO-1 induction. Three of the compounds (3,4-CQA, 3,5-CQA, and CGA) significantly up-regulated HO-1 in a concentration-dependent manner (Fig. 2) while *p*-CA had little effect.

### WEP induced early nuclear translocation of Nrf2 in NB1-RGB cells after UVA irradiation

**Fig. 3:**
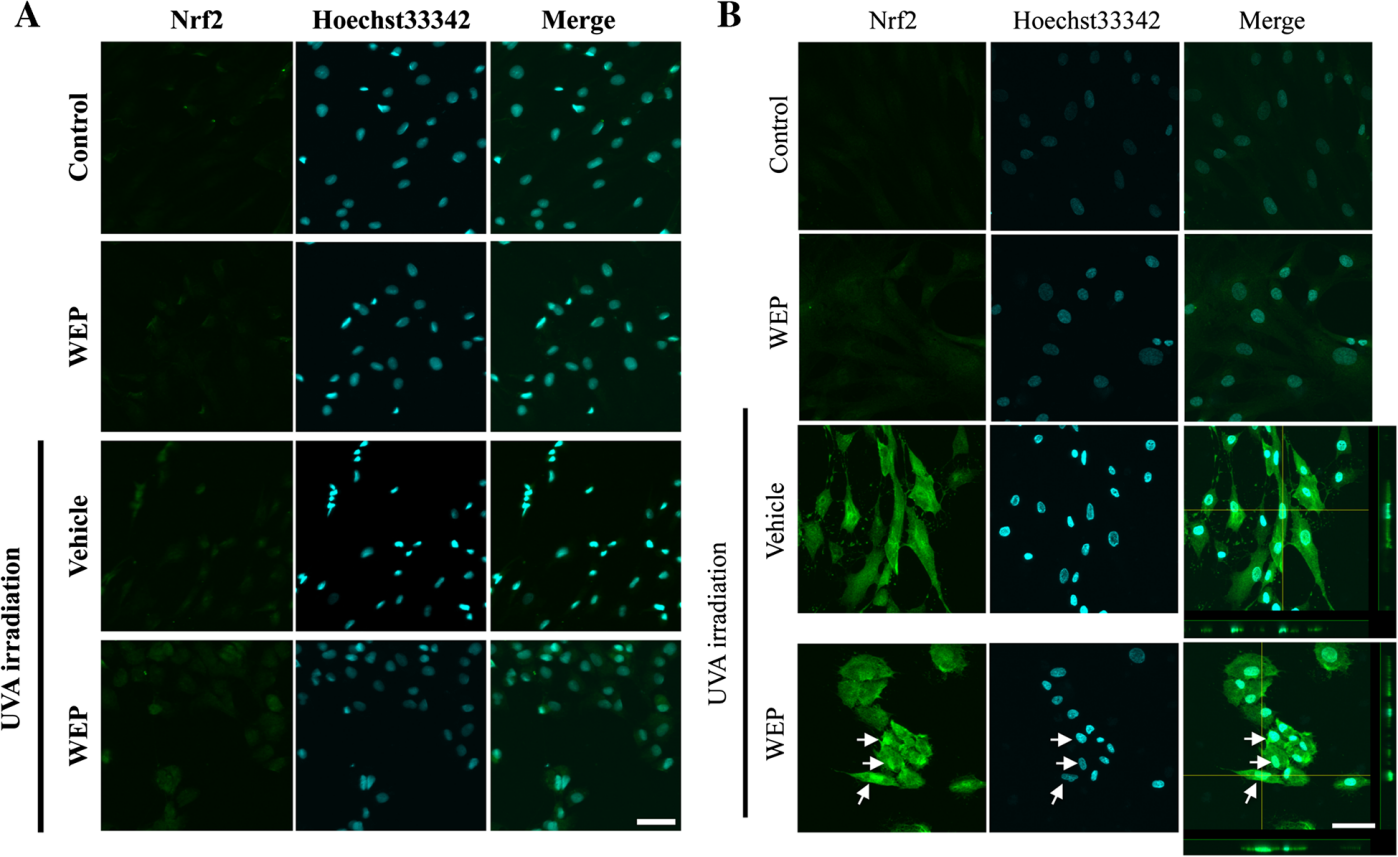
Nrf2 nuclear translocation was accelerated following treatment with WEP after UVA irradiation at 6 h after UVA irradiation. Immunostaining for Nrf2 in NB1-RGB cells at (**a**) 3, (**b**) 6 h after UVA irradiation showed changes induced by UVA irradiation and WEP treatment. Cells were fixed and stained with Hoechst33342 (blue) and anti-Nrf2 antibody (green). WEP was applied at a concentration of 30 μg/mL. White arrows indicate nuclear translocation of Nrf2; scale bar, 50 μm

We hypothesized that the WEP-induced HO-1 up-regulation at the early time points was attributable to the early nuclear translocation of Nrf2. To elucidate this, we conducted immunostaining of Nrf2 in NB1-RGB cells. Nrf2 expression slightly increased 3 h after UVA irradiation (Fig. 3a). At this time point, no apparent difference observed between UVA-treated group and WEP-UVA group.

Next, we investigated Nrf2 expression 6 h after UVA irradiation (Fig. 3b). At this time point, Nrf2 expression clearly increased. Moreover, at 6 h after UVA irradiation, Nrf2 nuclear co-localization (Hoechst33342) was observed in the WEP-pretreated group (white arrow, Fig. 3b). These results suggest that WEP pretreatment promoted the translocation of Nrf2 to the nuclei after UVA irradiation.

**Fig. 4:**
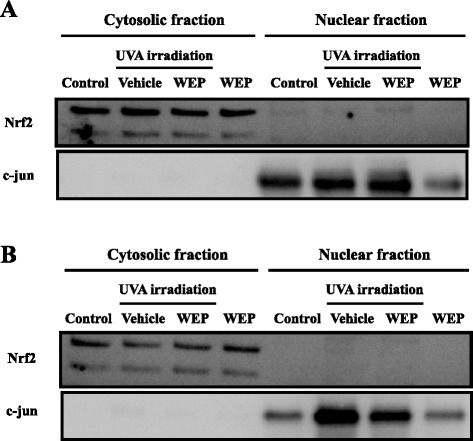
By western blot analysis, Nrf2 is almost not detected in nuclear fraction at 3, 6 h after UVA irradiation. Nrf2 expression level in cytosolic and nuclear fraction of NB1-RGB cells was evaluated using western blotting 3, 6 h after UVA irradiation. (**a**) 3 h after UVA irradiation, and (**b**) 6 h after UVA irradiation. At all time points, WEP was applied at a concentration of 30 μg/mL

Subsequently, we tried to measure nuclear Nrf2 upregulation with western blot method (Fig. 4a ~ b). Contrary to our expectation, between all groups, Nrf2 is only expressed in cytosolic fraction and almost not detected in nuclear fraction at 3 and 6 h after UVA irradiation. However, WEP-UVA group only slightly expressed Nrf2 in nuclear fraction at 3 h after UVA irradiation (Fig. 4a).

### WEP did not affect NQO1 expression

**Fig. 5:**
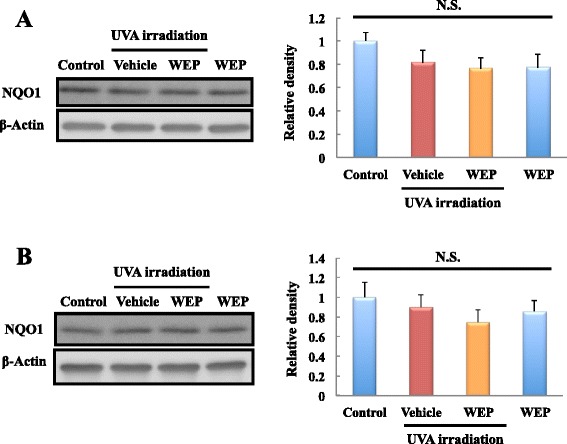
NQO1 is not changed at 3, 6 h after UVA irradiation. NQO1 expression level in NB1-RGB cells was evaluated using western blotting at each time point after initiating UVA irradiation (**a**) 3, (**b**) 6 h. At all time points, WEP was applied at a concentration of 30 μg/mL. Mean ± SEM (*n* = 5–6), NS means *P* > 0.05 vs. control (Tukey’s test)

We also investigated NQO-1 expression as the other antioxidant protein in addition to HO-1. Result of western blot analysis, NQO1 expression has not significant difference between all groups at 3 and 6 h after UVA irradiation (Fig. 5).

## Discussion

In this study, we demonstrated that WEP induced early up-regulation of HO-1 and nuclear translocation of Nrf2 following UVA irradiation. At 24 h after UVA irradiation, HO-1 expression markedly decreased in the group treated with WEP and UVA than the HO-1 expression in the group treated with UVA alone (Fig. 1d). This change may be induced by secondary effect of WEP. It is followed by acceleration of HO-1 up-regulation with decreasing ROS expression. This result indicates that total HO-1 expression remained unaltered after pretreatment with WEP, which only accelerates the antioxidative response. Nrf2 acts as an antioxidative transcriptional factor upon accumulation in the nuclei. Therefore, early nuclear translocation of Nrf2 after treatment with both WEP and UVA (Fig. 3) indicated that WEP activates the Nrf2/ARE pathway soon after UVA irradiation. Previous reports have shown that the Nrf2/Keap1 pathway plays an important role in protecting skin fibroblast against UVA-induced apoptosis [20, 21]. Therefore, it is highly possible that early up-regulation of HO-1 and nuclear translocation of Nrf2 contributed to the protective effect of WEP against UVA irradiation-induced cell death of human skin fibroblast, in our previous report [19].

Among the four major constituents of WEP, the diphenols significantly induced HO-1. Diphenol is oxidized to quinone under oxidative stress conditions and then reacts with the critical cysteine residues in Keap1 that are essential for its ubiquitin ligase substrate adaptor activity [22, 23]. Studies have reported various polyphenols with diphenol structures such as epigallocatechin gallate (EGCG) [24, 25], eckol [26, 27], and resveratrol [28, 29] that activate the Nrf2/ARE pathway. Moreover, 1,5-dicaffeoylquinic acid and 3-caffeoyl,4-dihydrocaffeoyl quinic acid were also reported to up-regulate HO-1 expression or induce Nrf2 nuclear translocation under certain oxidative stress conditions induced by oxygen and glucose deprivation/reperfusion or tert-butyl hydroperoxide exposure [30, 31]. They have a diphenol structure that is similar to that of 3,4-CQA, 3,5-CQA, and CGA. However, the underlying mechanism of HO-1 induction by these compounds remains unclear. For example, a previous report [30] indicated that 3-caffeoyl,4-dihydrocaffeoyl quinic acid regulates HO-1 expression via the phosphoinositide 3-kinase (PI3K)/Akt-Nrf2 signaling pathways but early up-regulation of HO-1 expression by WEP treatment was not inhibited by LY294002 (the PI3K/Akt pathway inhibitor) pretreatment (data not shown). Moreover, WEP and its main constituents did not induce HO-1 expression and nuclear translocation of Nrf2 in the absence of UVA irradiation. In contrast, the previous reports [30, 31] showed that the compounds induce the expression of these proteins without pre-induction of oxidative stress. Further investigation is required to elucidate these differences. In a previous study, we showed that *p*-CA, which does not possess a diphenol structure, also had protective effects [19]. Besides, in present study, we could not detect Nrf2 at 3 h after UVA irradiation (Figs. 3a and 4). NQO1, which the other downstream target protein of Nrf2, was not affected WEP pretreatment at 3, 6 h after UVA irradiation (Fig. 5). Based on these results, other mechanisms may also contribute to the protective effects of the main constituents of WEP. Since WEP acts as an HO-1 activator only after oxidative stress occurred in the cells, it can activate HO-1 only tissue under the pathological condition. These results suggest that WEP is useful for treatment of skin diseases as oxidation damaged portion-specific an HO-1 inducer.

## Conclusions

In conclusion, we demonstrated that WEP and its main constituents induce early up-regulation of HO-1 and nuclear translocation of Nrf2. Our findings indicated that WEP acts as an early inducer of HO-1 and a rapid activator of Nrf2 to protect against UVA-induced oxidative stress.

## Abbreviations

WEP: Water extract of propolis
EEP: Ethanol extract of propolis
3,4-CQA: 4-di-O-caffeoylquinic acid
3,5-CQA: 3,5-di-O-caffeoylquinic acid
p-CA: p-coumaric acid (p-CA)
EGCG: Epigallocatechin gallate
CGA: Chlorogenic acid
